# Evaluation of Satisfaction With a Secure, Connected Mobile App for Women in Assisted Reproductive Technology Programs: Prospective Observational Study

**DOI:** 10.2196/63570

**Published:** 2025-02-24

**Authors:** Pauline Plouvier, Romaric Marcilly, Geoffroy Robin, Chaymae Benamar, Camille Robin, Virginie Simon, Anne Sophie Piau, Isabelle Cambay, Jessica Schiro, Christine Decanter

**Affiliations:** 1 Departments of Assisted Reproductive Technologies and Fertility Preservation Jeanne de Flandre Hospital Lille France; 2 Inserm, CIC-IT 1403 F-59000 Lille France; 3 Univ. Lille, CHU Lille, ULR 2694 - METRICS : Évaluation des technologies de santé et des pratiques médicales F-59000 Lille France

**Keywords:** mobile apps, mHealth, mobile health, assisted reproductive technologies, evaluation, satisfaction, reproduction, fertility, ovarian stimulation, ease of use, usability, midwives, obstetrics, gynecology

## Abstract

**Background:**

Telemedicine has emerged rapidly as a novel and secure tool to deliver medical information and prescriptions. A secure, connected health care app (WiStim) has been developed in order to facilitate dialogue between patients and the medical team during an ovarian stimulation cycle for medically assisted reproduction (MAR).

**Objective:**

This study aimed to evaluate the patients’ and midwives’ levels of satisfaction with the connected mobile app.

**Methods:**

We conducted a prospective, observational, single-center study at Lille University Hospital, France. From May 1 to July 31, 2021, all women undergoing ovarian stimulation started to receive their treatment advice through the mobile app. A total of 184 women were included and they filled out the 30-item Usefulness Satisfaction and Ease-of-Use (USE) questionnaire, which examines the users’ opinions in 4 dimensions: usefulness, ease of use, ease of learning, and satisfaction. The women also answered a series of closed and open questions. The 5 midwives in our assisted reproductive technology center filled out the French version of the 10-item System Usability Scale (SUS) when the app was implemented and then after 3 and 6 months of use. We also performed semistructured interviews with the midwives.

**Results:**

Overall, 183 women using the app completed the questionnaire. None refused to use the app, and 1 withdrew from the study. The mean scores for the four USE dimensions were all significantly greater than 4, that is, the middle of the response scale. The women liked the app’s ease of use, the access to tutorial videos, and the reminders about appointments and treatments. In particular, the women liked to be able to (re)read the information; this reassured them, might have reduced the number of missed appointments and treatments, and made them more independent during the day, especially when they were working. Some of the women regretted the loss of direct contact with the midwife. The mean SUS score was 76 (SD 13.54) at the start of the study, 75 (SD 17.16) after 3 months, and 84 (11.21) after 6 months. According to the adjective rating scale, these scores corresponded to good usability for the app. After the requisite training and a familiarization period, the midwives reported that using the app saved them 2 hours a day. The mobile app enabled better transmission of information and thus probably helped to decrease treatment errors.

**Conclusions:**

The WiStim connected mobile app is one of the first reliable, secure apps in the field of MAR. The app reassured the patients during the ovarian stimulation. Women and the medical team considered that the app was easy and intuitive to use. Given the growth in demand for MAR programs and the medical team’s workload, the time savings provided by the app constitute a nonnegligible advantage.

## Introduction

In 2018, the European Society of Human Reproduction and Embryology’s 22nd report on medically assisted reproduction (MAR) in Europe highlighted a continuous increase in the number of treatment cycles and the broad range of techniques used [1].

Every day, around the world, thousands of women undergo hormone assays and ultrasound scans of the pelvis as part of their MAR program. These burdensome, complex procedures can generate stress and anxiety for the women and their partners [2-4]. The women consulting in MAR departments are young, active, and, in many cases, occupied by work and family activities. Furthermore, health care professionals have to deal with a growing administrative burden and increasing demand for MAR while still providing high-quality patient care and support.

A large number of “eHealth” smartphone apps are being developed [5-8]. WiStim is a secure mobile app created in 2016 to facilitate communication between MAR patients and medical teams. The app provides access to a variety of documents and media (eg, test results and tutorials on self-injection) and gives daily advice on treatment during the ovarian stimulation phase. The objectives are (1) secure communication with patients, (2) better traceability, (3) a lower frequency of treatment errors caused by poor understanding, and (4) time savings for the care team. At present, the patient has to pay a subscription fee; however, some hospitals are considering whether to subsidize or cover this fee.

During each in vitro fertilization, frozen embryo transfer, or intrauterine insemination cycle, the midwife calls the women daily in order to adjust the treatment, check on adherence, and answer any questions. These calls are time-consuming and can be replaced with a connected app.

The primary objective of the current study was to evaluate levels of satisfaction with the connected app, according to both the patients and health care professionals. The secondary objectives were to identify potential difficulties in the use of the app and to study any app-associated changes in the health care professionals’ practices and work organization.

## Methods

### Overview

We conducted a prospective, observational, single-center study in the MAR department at Lille University Hospital (Lille, France) from May 1 to July 31, 2021.

### Ethical Considerations

In line with the French legislation on human and social science surveys of routine clinical practice using anonymized personal data [9], approval by an independent ethics committee was neither required nor sought.

### Connected Mobile App

**Figure 1:**
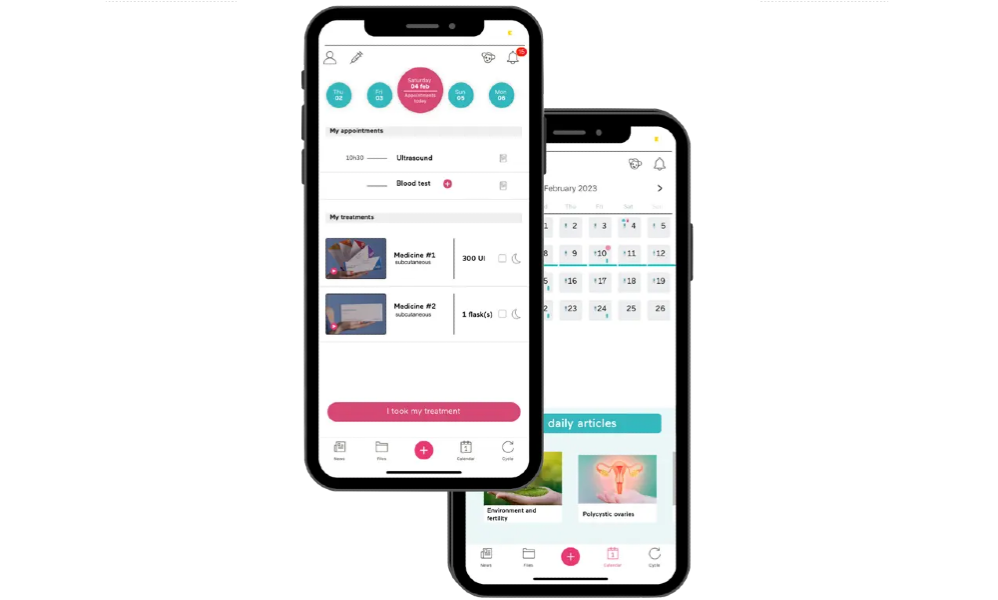
The mobile app for the patient.

The WiStim platform includes a mobile app for the patient (Figure 1) and a web-based management module for the medical team. The mobile app gives the patient access to advice on treatment, including the dose, the administration route, and any changes in the regimen. Tutorials on self-injections are also available. Every evening, the patient receives a reminder about her treatment and the date of her next appointment. The medical team sends information about the treatment and the next appointment through a secure web server (accredited by the French Ministry of Health) and is informed in real time about the woman’s treatment.

The app requires a monthly subscription. However, Lille University Hospital has chosen to pay this cost on the patient’s behalf during an initial test year.

### Patients

During a consultation, the gynecologist explained the app’s principles to the patient and gave the latter an information sheet explaining how to download, install, and use the app. The patient then installed the app on her mobile phone and created an account. Once the stimulation phase had started, the patient received treatment advice through the app.

At the end of the MAR procedure, the women filled out a questionnaire (Multimedia Appendix 1). The questionnaire’s closed questions concerned the type of MAR and the impact of using the app. The patient was able to write a comment for each closed question. Several open questions probed the woman’s opinion of the app and the app’s strengths and weaknesses. Next, the woman filled out the 30-item Usefulness Satisfaction and Ease-of-Use (USE) questionnaire, which measures the user’s feelings in four dimensions: usefulness, ease of use, ease of learning, and satisfaction (Multimedia Appendix 2) [10,11]. The participants were not paid for their participation in the study.

### Midwives

The five midwives in our MAR center filled out the French version of the System Usability Scale (SUS) when the app was implemented and then after 3 and 6 months of use [12]*.* The SUS (Multimedia Appendix 3) is a standardized, 10-item questionnaire for assessing the level of satisfaction with a technology’s usability. For each of the 10 items, the midwives were invited to rate their level of agreement on a 5-point Likert scale ranging from “strongly disagree” to “strongly agree.” We also performed semistructured interviews with the midwives 6 months after the implementation of the app in order to evaluate their opinion of the connected app, identify any changes in their work organization, and assess the app’s acceptability (as defined in the unified theory of acceptance and use of technology [UTAUT]) [13]. The interviews were audio-recorded and transcribed.

### Statistical Analyses

Statistical analyses were performed using Jamovi software (Jonathon Love, Damian Dropmann, and Ravi Selker, version 2.2.5, 2021), and thematic analyses of qualitative data were performed using QualCoder 3.0 (C Curtain). Data on the women’s characteristics were used to describe the sample and perform subgroup analyses. The women’s answers to the closed questions were analyzed using descriptive statistics, and the comments were analyzed qualitatively in order to highlight recurrent themes. Emerging themes in the qualitative analyses were not quantified; the goal was to understand the variety of the user’s points of view. The data on the USE items and dimensions were analyzed with descriptive and inferential statistics. The threshold for statistical significance was set to *P*<.05. The results of the various questionnaires were compared in order to identify similarities and differences between the answers.

The SUS satisfaction score ranges from 1 to 100. Perceived satisfaction and usability are considered to be good when a score of 75 or more is obtained. To qualify the level of satisfaction, the mean SUS score was assessed against Aaron et al [14] grade scale and adjective rating scale. Semantic units (ie, sets of words representing the same idea) were extracted from the interviews with the midwives. An ergonomist attributed each semantic unit to one of the following themes: the opinion of the app, changes in practice, and dimensions of UTAUT acceptability (facilitating conditions, effort expectancy, social influence, and performance expectancy). Within each theme, several subthemes were developed to represent the semantic units’ diversity.

## Results

### Patients

#### Overview

During the recruitment period, 250 paper questionnaires were randomly distributed, and 184 patients responded (intrauterine insemination: 12%, in vitro fertilization: 73%, egg donation: 2%, frozen-thawed embryo transfer: 9%, fertility preservation: 4%). A total of 91 respondents had already been followed up by phone before the implementation of the app, 90 were monitored with the connected app alone, and 3 did not answer this item in the questionnaire. One woman discontinued use of the app prematurely, preferring direct contact with a midwife, and 2 women failed to complete the USE questionnaire.

The mean scores for the four USE dimensions were all significantly greater than 4, that is, the middle of the response scale (usefulness, n=5.66, *t*_180_=13.4, *P*<.001; ease of learning, n=6.25, *t*_178_=18, *P*<.001; ease of use, n=5.96, *t*_180_=16, *P*<.001; satisfaction, n=5.90, *t*_177_=15.4, *P*<.001). The scores did not appear to be influenced by whether or not the women had been followed up by phone before the use of the app.

This good level of perceived usability was corroborated by our analysis of the women’s comments and answers to the open questions. In all, 172 women described what they liked about the app. The app was seen as being easy to use in a guided, stepwise manner (“simple to use,” “intuitive,” and “clear”). Another reason for liking the app was its match with the women’s needs and activities. The women mentioned the gain in efficiency linked to the app’s centralization of information on treatment, follow-up, and appointments.

**Figure 2:**
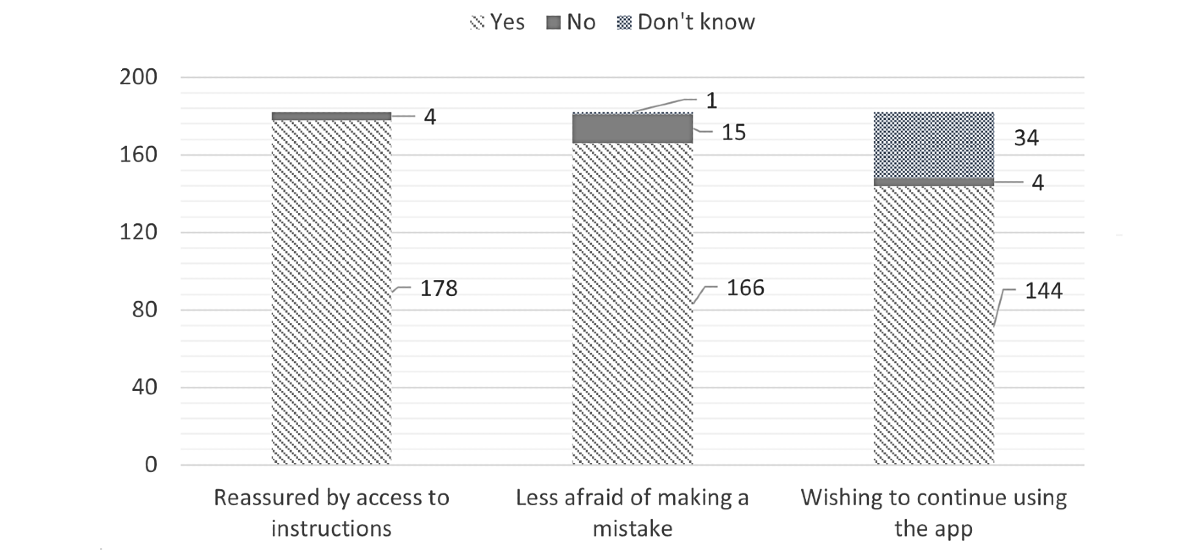
Answers to the following closed questions: “Does access to advice in the mobile app reassure you?” “Have you felt less stressed since you starting using the mobile app?” “Would you like to continue using the mobile app?”.

A total of 178 (97.8%) of the 182 respondents considered that the ability to access advice through the app was reassuring (Figure 2). The participants, having initially been followed up by phone, stated that they felt surer about the advice with the app because the instructions had to be rapidly written down during the phone call; with the app, the advice was available round the clock. The women also liked the additional information (eg, how to perform an injection) and the automatic reminders about appointments and treatments. A total of 166 (91.7%) of the 181 respondents considered that with the app, they were less stressed about making a treatment mistake. The participants liked the independence that the app provided; they no longer had to wait for the phone call from the midwife; previously, the call could come at any time in the afternoon.

Overall, 34 women did not reply to the question about wanting to continue to use the connected mobile app or not. 97.3% of the respondents said that they would be annoyed if they had to stop using the app (Figure 2). The 4 participants, who said they would feel relieved if they had to stop using the app, had all initially been followed up by phone and preferred direct communication with the medical team.

Some weaknesses were mentioned: appointment errors, the lack of tutorials about some older models of self-injector pens, and late treatment reminders (ie, given after the scheduled time). Certain women expressed the need for direct interaction with the team of midwives via a chat function; the MAR department had decided not to offer this function because the women had already been given an email address for any questions.

A total of 53 (58.2%) of the 91 women who had initially been followed up by phone did not perceive any change in how they managed their treatment since the implementation of the app. In contrast, 38 (41.7%) women stated that the app modified the management of their treatment; they highlighted the lower mental burden, the easy, permanent access to information, the reassuring nature of the treatment reminders and app reminders, and the fact their day no longer had to be organized around the call from the midwife.

#### Midwives

**Figure 3:**
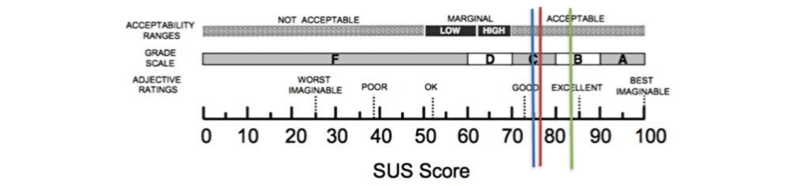
The mean System Usability Scale (SUS) score at the start of the study (76, SD 13.54) and after 3 months (75, SD 17.16) and 6 months (84, SD 11.21).

The five midwives working in the MAR department at the time of the study filled out the SUS and were interviewed. The mean age was 43.6, and the midwives had been working in the department for an average of 6 years. The mean SUS score was 76 at the start of the study (n=4 respondents), 75 after 3 months (n=5), and 84 after 6 months (n=4; Figure 3). According to Aaron et al [14] adjective rating scale, these scores corresponded to good usability for the app.

A total of 207 semantic units were extracted from the semistructured interviews and analyzed. Even though the midwives stated that they needed some time to learn how to use the app and that the use of the app reduced the level of human contact with the women, they were generally very satisfied. The midwives found the interface pleasant to use and considered that the app was useful for the women and for the nurses who visited the women at home on a routine basis; the app modernized the midwives’ practices.

During the interviews with the midwives, 3 of the 4 UTAUT dimensions emerged: facilitating conditions, effort expectancy, and performance expectancy. The social influence dimension did not emerge.

### Facilitating Conditions

The midwives said that for the installation of the app, some IT equipment (computers) had been purchased and that they had attended training sessions on the use of the app. The midwives emphasized the good availability and responsiveness of the WiStim team when questions arose.

### Effort Expectancy

The midwives viewed the app as a rather simple-to-use tool once they had been trained in its use. The app’s different functions enable them to consult the patients’ data, communicate with them, plan their appointments, and monitor their adherence to treatment.

Given that the app had not been integrated into the hospital information system at the time of the study, the midwives could not enter appointments into the hospital information system’s diary from the app or vice versa; hence, to make appointments, they had to work on two IT tools in parallel. Even though the app had a simple-to-use interface, the fact that it was a web-based tool meant that the transitions were sometimes slow. Hence, data sometimes had to be entered twice: once on paper and then on the app.

### Performance Expectancy

Use of the app was associated with a fall in the number of daily calls from midwives to patients. Nevertheless, the midwives continued to phone the women when the medical team had decided to stop the treatment or when the women had not ticked the boxes for adherence to treatment. This enabled them to optimize the dialogue by phone. Certain women continued to call or email the midwives regardless, to check that they had correctly understood the treatment procedures and advice specified by the app or to change an appointment. The midwives replied to these questions by email or by phone. The frequency of these requests tended to fall over time, as the midwives and the patients became more familiar with the app.

In order to ensure that the women were using the app correctly, the midwives helped some of them (especially those who did not understand or speak French sufficiently well) to install the app and explained how to use it. Furthermore, the advice stored in the app can be presented in several languages.

The decrease in the number of phone calls freed up an average of 2 hours per day, which enabled the midwives to perform other tasks (consultations, patient education sessions, etc).

## Discussion

### Principal Findings

On an annual basis, our center performs around 1400 oocyte retrieval procedures, 900 frozen embryo transfers, and 700 intrauterine inseminations. This corresponds to 30 to 50 women per day seen for ultrasound scans and hormone assays, and the volume of activity is increasing. Until now in France, MAR has only been available to heterosexual couples on medical indication. The law on bioethics, to be promulgated in 2021, extends MAR to female couples and single women. Activity in MAR centers has therefore risen sharply. Use of the connected mobile app was associated with a considerable decrease in the number of phone calls during the day. This resulted in significant time savings for the medical team, who were therefore able to perform other tasks. The women received their treatment advice in a secure manner, which had not been the case previously. The women felt reassured and less stressed about making treatment mistakes.

We did not question the partners, but in the open-ended questions, some of them said that they were reassured to be able to read the treatment instructions and felt more involved in the care compared with the phone call.

At present, it is difficult to quantify the extent of treatment errors during an ovarian stimulation program. Till date, no study has been published in the literature on this subject. Nevertheless, along with the treatment reminders, the fact that the woman’s prescription is given in writing in the app and can be accessed at any time (and in several languages, if required) probably helped to reduce forgetfulness and treatment administration errors. This traceability is essential for secure communication between patients and professionals. Barrière et al [15] conducted an observational, real-life, longitudinal study involving 488 patients from 28 infertility centers in France to evaluate patient–infertility care provider relationships and communication in Assisted Reproductive Technology centers and investigate whether the quality of the care provided had an impact on patient adherence to treatment and monitoring protocols. They showed that even when patient-physician relationships appear to be satisfactory, patient miscomprehension and noncompliance during infertility treatment may be underestimated, and improvements in communication are also required.

Several studies [16,17] and reviews of the literature [18-20] have shown that stress is a major risk factor for impaired quality of life and a poor experience of infertility treatment. It also exposes patients to the risk of abandoning assisted reproduction procedures, thereby reducing their chances of pregnancy. It is therefore conceivable that mobile apps can reassure patients undergoing infertility treatments and consequently reduce the stress inherent in these procedures, as suggested in a recent review [21]. Further studies using standardized questionnaires and comparing the anxiety levels of the app users with those of nonusers could answer this hypothesis. The risk of dropping out of infertility management procedures could also be compared between these two groups.

Over the last decade, huge progress has been made in information and communication technology. “eHealth” and smart (connected) devices have brought together services that facilitate communication with the patient and thus improve the overall quality of care (teleconsultations, electronic health records, smart eHealth apps, etc) [22]*.* Telemedicine enables medical data to be shared with the patient or between health care professionals. Many connected health apps have been developed – notably in the fields of radiology and cardiology [23]. A recent study showed that connected health technologies can facilitate access to cancer care and improve the patient’s psychological well-being and quality of life [24]. Recently, connected apps have been developed for use in gynecology and obstetrics [25]. There are probably other connected apps in the world in the field of fertility, but to date and to our knowledge, there are very few publications on this subject. Boivin et al [26] were interested in the development of a mobile app (MediEmo) to provide support during medically assisted reproduction. MediEmo is an app combining patient medication diary management and ease of integration into clinic systems with emotional support and data capture. They showed that 98% of patients expressed willingness to use the app, and almost 80% did so. Thus, the development of smartphone apps can contribute to fertility care and should be encouraged.

The profile of women in MAR programs is particularly suitable for this type of development: they are young and active and they all use a smartphone.

The protection of the patients’ personal data is a crucial aspect in the development of connected health technologies [27,28]. WiStim complies with the European Union’s General Data Protection Regulation, and the app’s host is accredited by the French Ministry of Health.

One limitation is that the app is currently fee-based, although various funding options are being considered. In the present case, our hospital paid for the subscription to the app, and so it was cost-free for the users. However, the hospital’s finance department is currently carrying out a medical-economic survey to assess the profitability of financing the app in relation to the time saved by the midwives. In fact, the midwife who used to call patients back can now do consultations instead.

Another limitation is the relatively small number of questionnaires analyzed and the fact that this was a single-center study. These are preliminary results, and they pave the way for further investigations in a larger population. Indeed, a multicenter study, with more questionnaires and over a longer period would be desirable to confirm these data.

Moreover, the questionnaires were distributed as soon as the app was installed in the department, so a learning curve was necessary for both patients and professionals. Overall satisfaction is probably higher now that the tool is better understood. Till now, all our patients have used the app.

### Conclusion

Our results showed that the use of the connected health care app reassured women during the ovarian stimulation phase of a MAR program. Both the women and the medical team considered that the app was easy and intuitive to use. The information on treatment was sent in a secure manner and could be accessed around the clock by the patient. Connected health care apps have been developed widely in recent years. WiStim is one of the first reliable, secure apps in the field of MAR. Given the growth in demand for the MAR program and the medical team’s workload, the time savings provided by the app constitute a nonnegligible advantage.

## Appendix

Multimedia Appendix 1 Patient questionnaire.

Multimedia Appendix 2 The Usefulness Satisfaction and Ease-of-Use questionnaire.

Multimedia Appendix 3 System usability scale.

## Abbreviations

MAR: medically assisted reproduction
SUS: System Usability Scale
USE: Usefulness Satisfaction and Ease-of-Use
UTAUT: unified theory of acceptance and use of technology
