# Supplementary material for: Evaluation of Satisfaction With a Secure, Connected Mobile App for Women in Assisted Reproductive Technology Programs: Prospective Observational Study
Source: JMIR Hum Factors. 2025 Feb 24;12:e63570. doi: 10.2196/63570 (PMC11894345; doi:10.2196/63570)
Supplement: Multimedia Appendix 1 [file humanfactors_v12i1e63570_app1.docx]

Family name and first name:

Are you been followed up for:

□ artificial insemination

□ IVF/ICSI

□ frozen embryo transfer

□ fertility preservation

□ oocyte donation?

Is this your first cycle?

□ Yes

□ No

1/ In your opinion, what are the strengths of the WiStim application?

2/ What are its weaknesses?

3/ Did the WiStim application change something in the way you managed your treatment?

□ Yes

□ No

If yes, why was that?

4/ If you had to stop using the WiStim application tomorrow, would you be relieved or annoyed?

□ Relieved

□ Annoyed

Why would that be?

5/ Do you feel that having access to written advice in the application is reassuring?

□ Yes

□ No

6/ Are you less stressed about making treatment mistakes?

□ Yes

□ No

Other comments:
