# Supplementary material for: Evaluation of Satisfaction With a Secure, Connected Mobile App for Women in Assisted Reproductive Technology Programs: Prospective Observational Study
Source: JMIR Hum Factors. 2025 Feb 24;12:e63570. doi: 10.2196/63570 (PMC11894345; doi:10.2196/63570)
Supplement: Multimedia Appendix 2 [file humanfactors_v12i1e63570_app2.docx]

**The Usefulness Satisfaction and Ease-of-Use questionnaire**

|  | Strongly disagree |  |  |  |  |  | Strongly agree | NA |
| --- | --- | --- | --- | --- | --- | --- | --- | --- |
| It helps me be more effective. | □ | □ | □ | □ | □ | □ | □ | □ |
| It helps me be more productive. | □ | □ | □ | □ | □ | □ | □ | □ |
| It is useful. | □ | □ | □ | □ | □ | □ | □ | □ |
| It gives me more control over the activities in my life. | □ | □ | □ | □ | □ | □ | □ | □ |
| It makes the things I want to accomplish easier to get done. | □ | □ | □ | □ | □ | □ | □ | □ |
| It saves me time when I use it. | □ | □ | □ | □ | □ | □ | □ | □ |
| It meets my needs. | □ | □ | □ | □ | □ | □ | □ | □ |
| It does everything I would expect it to do. | □ | □ | □ | □ | □ | □ | □ | □ |
| It is easy to use. | □ | □ | □ | □ | □ | □ | □ | □ |
| It is simple to use. | □ | □ | □ | □ | □ | □ | □ | □ |
| It is user friendly. | □ | □ | □ | □ | □ | □ | □ | □ |
| It requires the fewest steps possible to accomplish what I want to do with it. | □ | □ | □ | □ | □ | □ | □ | □ |
| It is flexible. | □ | □ | □ | □ | □ | □ | □ | □ |
| Using it is effortless. | □ | □ | □ | □ | □ | □ | □ | □ |
| I can use it without written instructions. | □ | □ | □ | □ | □ | □ | □ | □ |
| I don't notice any inconsistences as I use it. | □ | □ | □ | □ | □ | □ | □ | □ |
| Both occasional and regular users would like it. | □ | □ | □ | □ | □ | □ | □ | □ |
| I can recover from mistakes quickly and easily. | □ | □ | □ | □ | □ | □ | □ | □ |
| I can use it successfully every time. | □ | □ | □ | □ | □ | □ | □ | □ |
| I learned to use it quickly. | □ | □ | □ | □ | □ | □ | □ | □ |
| I easily remember how to use it. | □ | □ | □ | □ | □ | □ | □ | □ |
| It is easy to learn to use it. | □ | □ | □ | □ | □ | □ | □ | □ |
| I quickly became skillful with it. | □ | □ | □ | □ | □ | □ | □ | □ |
| I am satisfied with it. | □ | □ | □ | □ | □ | □ | □ | □ |
| I would recommend it to a friend. | □ | □ | □ | □ | □ | □ | □ | □ |
| It is fun to use. | □ | □ | □ | □ | □ | □ | □ | □ |
| It works the way I want it to work. | □ | □ | □ | □ | □ | □ | □ | □ |
| It is wonderful. | □ | □ | □ | □ | □ | □ | □ | □ |
| I feel I need to have it. | □ | □ | □ | □ | □ | □ | □ | □ |
| It is pleasant to use. | □ | □ | □ | □ | □ | □ | □ | □ |
