# Supplementary material for: Evaluation of Satisfaction With a Secure, Connected Mobile App for Women in Assisted Reproductive Technology Programs: Prospective Observational Study
Source: JMIR Hum Factors. 2025 Feb 24;12:e63570. doi: 10.2196/63570 (PMC11894345; doi:10.2196/63570)
Supplement: Multimedia Appendix 3 [file humanfactors_v12i1e63570_app3.docx]

**System Usability Scale**

|  | Strongly disagree |  |  |  | Strongly agree |
| --- | --- | --- | --- | --- | --- |
| I think that I would like to use this system frequently. | □ | □ | □ | □ | □ |
| I found the system unnecessarily complex. | □ | □ | □ | □ | □ |
| I thought the system was easy to use. | □ | □ | □ | □ | □ |
| I think that I would need the support of a technical person to be able to use this system. | □ | □ | □ | □ | □ |
| I found the various functions in this system were well integrated. | □ | □ | □ | □ | □ |
| I thought there was too much inconsistency in this system. | □ | □ | □ | □ | □ |
| I would imagine that most people would learn to use this system very quickly. | □ | □ | □ | □ | □ |
| I found the system very cumbersome to use. | □ | □ | □ | □ | □ |
| I felt very confident using the system. | □ | □ | □ | □ | □ |
